# Protocol of a prospective community-based study about the onset and course of depression in a nationally representative cohort of adults in China: the China Depression Cohort Study-I

**DOI:** 10.1186/s12889-023-16542-6

**Published:** 2023-08-24

**Authors:** Xuting Li, Yusheng Tian, Michael R. Phillips, Shuiyuan Xiao, Xiaojie Zhang, Zongchang Li, Jun Liu, Lingjiang Li, Jiansong Zhou, Xiaoping Wang

**Affiliations:** 1 Department of Psychiatry, National Clinical Research Center for Mental Disorders, The Second Xiangya Hospital, Central South University, Changsha, China; 2 Clinical Nursing Teaching and Research Section, The Second Xiangya Hospital, Central South University, Changsha, China; 3 Shanghai Mental Health Center, Shanghai Jiao Tong University School of Medicine, Shanghai, China; 4 Department of Radiology, The Second Xiangya Hospital, Central South University, Changsha, China

**Keywords:** Depression, Prospective, Cohort study, Community-based, China

## Abstract

**Background:**

Depression is the second most important cause of disability worldwide. Reducing this major burden on global health requires a better understanding of the etiology, risk factors, and course of the disorder. With the goal of improving the prevention, recognition, and appropriate management of depressive disorders in China, the China Depression Cohort Study will establish a nationally representative sample of at least 85,000 adults (the China Depression Cohort Study-I) and 15,000 middle school students (the China Depression Cohort Study-II) and follow them over time to identify factors that influence the onset, characteristics, and course of depressive disorders. This protocol describes the China Depression Cohort Study-I.

**Methods:**

A multistage stratified random sampling method will be used to identify a nationally representative community-based cohort of at least 85,000 adults (i.e., ≥ 18 years of age) from 34 communities in 17 of mainland China’s 31 provincial-level administrative regions. Baseline data collection includes 1) demographic, social and clinical data, 2) diagnostic information, 3) biological samples (i.e., blood, urine, hair), 4) brain MRI scans, and 5) environmental data (e.g., community-level metrics of climate change, air pollution, and socio-economic characteristics). Baseline findings will identify participants with or without depressive disorders. Annual reassessments will monitor potential risk factors for depression and identify incident cases of depression. Cox Proportional-Hazards Regression, Network analysis, Disease trajectory modelling, and Machine learning prediction models will be used to analyze the collected data. The study’s main outcomes are the occurrence of depressive disorders; secondary outcomes include adverse behaviors (e.g., self-harm, suicide), the recurrence of depression and the incidence other mental disorders.

**Discussion:**

The China Depression Cohort Study-I will collect a comprehensive, nationally representative set of individual-level and community-level variables over time. The findings will reframe the understanding of depression from a ‘biology-psychology-society’ perspective. This perspective will improve psychiatrists’ understanding of depression and, thus, promote the development of more effective subgroup-specific antidepressant drugs and other interventions based on the new biomarkers and relationships identified in the study.

**Trail registration:**

The protocol has been registered on the Chinese Clinical Trial Registry (No. ChiCTR2200059016).

## Introduction

### Background

Depressive disorder was one of the top ten causes of disability-adjusted life-years (DALYs) lost worldwide in the 2019 Global Burden of Disease report [1]. The estimated global age-standardized prevalence of depressive disorders in 2019 was 3.1 ~ 3.8% [2]. Depression can occur at any age; the lifetime risk of depression is 15 ~ 18% [3]. Depression is a recurring disorder that is often treatment-resistant; 20 ~ 40% of patients treated for a major depressive disorder do not achieve remission after two or more courses of treatment with different antidepressants [4, 5]. Depression is an important risk factor for fatal and non-fatal suicidal behavior [6].

Reducing the global burden of depressive disorders requires a detailed understanding of the risk factors, biomarkers, course, and treatment responsiveness of the different subtypes of depression. Effectively using these findings to improve outcomes also requires detailed information about the community-specific identification, availability of treatment options, and care-seeking of persons with depression. A wide range of pathophysiological models and pathogenetic mechanisms have been proposed: the monoamine hypothesis, hypothalamic–pituitary–adrenal axis changes; inflammation; neuroplasticity and neurogenesis; structural and functional brain changes; genes; environmental milieu; and epigenetics (gene-environment interactions) [3]. No single model or mechanism can explain the complex interaction between external environmental factors and individual biological characteristics that regulates the onset and course of depressive disorders. Recent studies suggest that a variety of community-level environmental factors, such as air pollution [7, 8], green space exposure [9] and climate change [10], have significant associations with the onset and course of depression. But the mechanisms that regulate the interaction of these community-level factors with individual-level factors remain unknown [11]. Large longitudinal datasets that include community-level environmental factors and individual biological samples of community residents will be needed to clarify this relationship and, thus, understand the etiology of depression from the population level. We hypothesize that depressive disorders result from dynamic interactions among biological factors (e.g., genetic susceptibility), psychological characteristics (e.g., personal traits) and social-environmental factors (e.g., economic conditions, life events and climate change).

Understanding the dynamic relationships between environmental and individual factors that underpin depressive states require multi-faceted, longitudinal studies of large, representative samples. Such cohort studies are the best way to identify the incidence and natural history of diseases [12] and to simultaneously assess associations between multiple exposures and outcomes [13]. Several large cohort studies of depression have been reported, some following cohorts of community-based residents and others following cohorts of individuals with a history of depression. 1) A prospective birth cohort study in Brazil identified robbery in the prior 12 months as a risk factor for major depressive disorders [14]. 2) A population-based database from the UK Biobank found that patients with depression were more likely to have elevated levels of C-reactive protein (CRP), indicating low-grade inflammation; however, this relationship was no longer statistically significant after adjusting for smoking and BMI, suggesting that the presumed genetic contribution to increased inflammation in depression may be regulated by eating and smoking habits rather than a genetic predisposition [15]. 3) The Netherlands Study of Depression in Older Persons (NESDO) [16] followed a cohort of individuals with depression and reported that older age, early onset, severity of depressive symptoms, anxiety symptoms, comorbid anxiety disorder, fatigue, and loneliness were independent risk factors for late-life depression [17]. 4) The Netherlands Mental Health Survey and Incidence Study (NEMESIS) [18–20] followed a cohort of community residents 18–64 years of age and found that 12% of the subthreshold cases developed a depressive disorder during three years of follow-up. 5) The Australian Genetics of Depression Study collected questionnaire data and saliva samples from 20,689 participants (88% of whom met criteria for a lifetime depressive episode) to investigate the genetic architecture of depression and common comorbidities [21]. 6) The Uppsala Longitudinal Adolescent Depression Study in Sweden and the LIFE Child Depression study in Germany followed children and adolescents with depression until adulthood [22, 23]. And 7) some small-sample cohort studies investigated risk factors for depression in children with cancer [24], and in adolescents with sleep disorder [25].

Cohort studies about depression in China include small-sample studies focused on perinatal depression [26, 27] and large-sample studies that piggy-back on cohorts collected for other reasons such as The China Hainan Centenarian Cohort Study (CHCCS) and the China Kadoorie Biobank (CKB) study [28, 29]. These prior studies in China concentrated on the consequences of depression, not on the causes of depression. There is no nationally representative longitudinal cohort study of depression in China.

Most of the currently available cohort studies about depression have serious limitations. 1) The studies often include patients with depression at baseline, so they tend to focus on the outcomes of treatment rather than on the causes of the disorder. 2) Very few studies consider community-level environmental factors such as local impacts of climate change, level of urbanization, socio-economic status of the community, availability and quality of health services, and patterns of health-seeking behavior for mental disorders. 3) None of the available studies consider the role of dynamic interactions between community-level environmental factors and the characteristics of individual community members (such as genetic susceptibility, neural circuits and intestinal flora) on the onset and course of depressive disorders. And 4) few cohort studies propose or test predictive models about the mechanisms that result in depressive disorders.

The China Depression Cohort Study will address most of these limitations. It will recruit a representative community-based cohort of at least 85,000 adult individuals (the China Depression Cohort Study-I) and a school-based cohort of at least 15,000 middle school students 12 years of age or older (the China Depression Cohort Study-II). This report is a detailed protocol of The China Depression Cohort Study-I (hereafter referred to as “this study”).

### Aims

The primary objective of this study is to explore the etiology of depression from the ‘biology-psychology-society’ perspective. The specific aims are to 1) identify risk factors that influence the onset, severity, course and relapse of depressive disorders; 2) characterize distinct subtypes of depression; and 3) describe the mechanisms that regulate the interactions of environmental, social, psychological, and biological factors that are associated with the onset and course of depressive disorders.

## Methods and analyses

### Study design

**Fig. 1:**
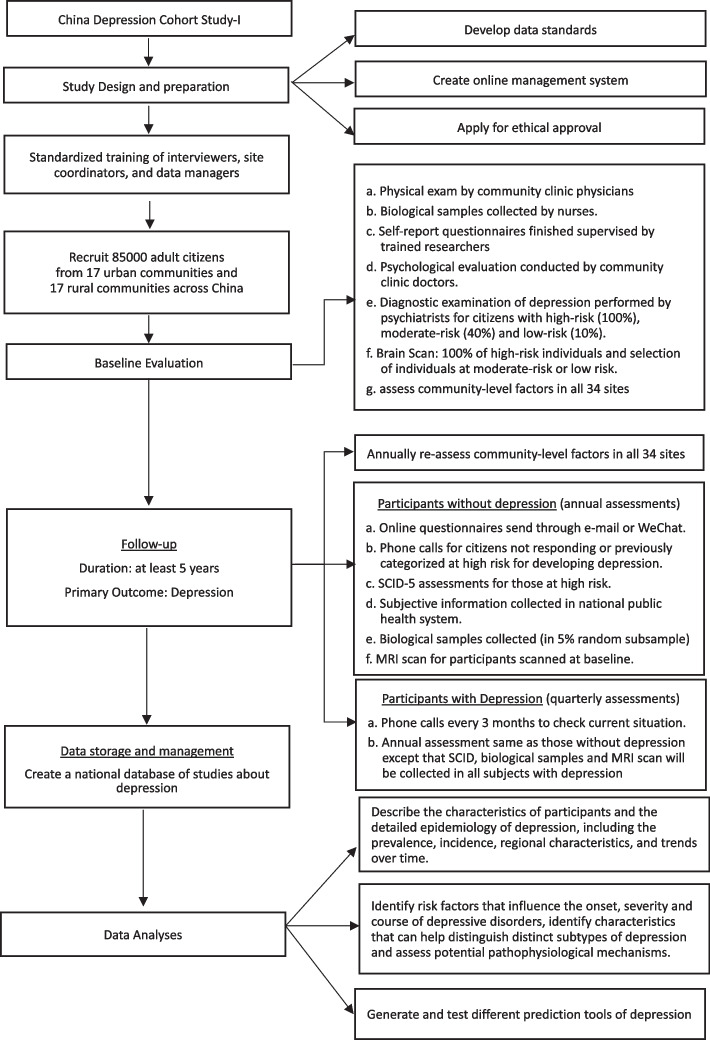
Organization of the China Depression Cohort Study-I

This is a longitudinal prospective cohort study of a representative sample of community-dwelling residents in mainland China. The onset of episodes of depression in participants will be identified and the course of identified episodes will be monitored closely. Figure 1 shows the overall design of the project.

### Study population

#### Sample criteria

Chinese citizens 18 years of age or older currently living in the selected communities who are registered permanent residents of the community (i.e., whose formal household registration is in the community) will be potential participants. Individuals who are transient residents, citizens of foreign countries, and persons living in institutional settings (chronic-care hospitals, boarding schools and universities, factories, prisons, etc.) will be excluded from the sampling frame.

#### Sampling method

We will use a multistage stratified random sampling method to identify 17 urban and 17 rural primary sampling sites in 17 of the 31 province-level administrative regions (hereafter, ‘provinces’) in mainland China. The 31 mainland provinces were first stratified by China’s seven administrative regions (Northeast, North, Northwest, East, Central, South and Southwest China) and then specific provinces from all seven geographic regions were selected to be nationally representative of the economic level and population size of the 31 different provinces. The 17 selected provinces included Heilongjiang and Liaoning from Northeast China; Beijing, Hebei, Tianjin, and Shanxi from North China; Gansu, Shanxi and Xinjiang from Northwest China; Zhejiang and Shanghai from East China; Henan, Hunan and Hubei from Central China; Guangdong from South China; and Sichuan and Chongqing from Southwest China.

Within each selected province, the second stage of sampling relied on China’s Disease Surveillance Points (DSP) System, a national representative sampling frame developed by the National Health Commission of the People’s Republic of China starting in 1978 that uses a stratified cluster random sampling method to select surveillance points (i.e., geographic areas that typically includes multiple communities or neighborhoods) around the country [30]. By 2021, a total of 605 points from all 31 provinces were included in this system, with a combined population of over 300 million individuals [30]. The disease surveillance points system provides a nationally representative cohort that has been widely used in national studies about the mortality and morbidity of various diseases [31, 32]. The number of DSP in the 17 selected provinces ranged from 7 (in Tianjin, Beijing and Shanghai) to 27 (in Heilongjiang). The DSP in each province were first stratified as urban or rural (based criteria specified by the National Bureau of Statistics) and then one urban and one rural DSP was selected for each province, resulting in a total 34 DSP. The criteria for selecting DSP within a province included 1) having a mean age of community members similar to that of all urban residents or all rural residents in the province; 2) having qualified mental health providers who were willing to participate in the management of the project; and 3) availability of MRI equipment needed to conduct the imaging part of the assessment.

Each DSP covered a variable number of specific communities, so the third stage of sampling was to select a single community within each of the 34 DSP where the project will be conducted. The criteria for selecting the 34 specific communities (‘primary sampling units’) included 1) having a mean age of community members similar to that of the entire DSP; 2) having a population of at least 3250 adults who are permanent residents (the minimum estimated sample required to enrolled 2500 adults); and 3) having local health institutions with sufficient human resources to perform the enrollment and follow-up procedures that are willing to participate in the project.

All permanent residents of the target communities meeting the inclusion and exclusion criteria will be eligible for inclusion in the cohort. Permanent residents meeting these criteria will be identified from the registers of residents in community health centers (which list virtually all permanent community members). Initially 3250 eligible individuals will be randomly selected from the lists of residents: after listing all adult eligible residents the total number of listed residents is divided by 3250 and the resultant integer and its fractional component (x.yy) are used to assess the proportion of the total list from which each x^th^ individual should be selected and the proportion of the total list from which each (x + 1)^th^ individual should be selected to arrive at a total sample of 3250. As many of these individuals as possible will be recruited into the project, even if the number of enrollees exceeds the minimum required sample of 2500 individuals. If contacting the 3250 eligible individuals does not result in enrolling 2500 individuals, an addition number of individuals from the resident list who were not selected the first time will be randomly selected in the same manner and invited to participate (considering the potential non-response rate of 30%, the additional number will be defined as 1.3 times of the differences between 2500 and actual number); this process will be repeated as needed until 2500 individuals have been enrolled. Reasons for non-enrollment and for post-enrollment drop-out will be described and the potential effects of selective enrollment and dropouts on the representativeness of the sample will be assessed by comparing the demographic characteristics of enrolled individuals with those of individuals who are selected for recruitment but not enrolled and of persons who drop out after enrollment with those of individuals who continue in the cohort.

#### Sample size

Based on a previous cross-sectional epidemiological study, the prevalence of major depressive disorder among adults in China was 2.1% [33]. Using version 15.0 of the Power Analysis & Sample Size (PASS) package, a sample size of 79,986 is required to identify a condition with a prevalence of 2.1% with a 95% confidence interval of 2.0% to 2.2%. We conservatively decided on a total target cohort of 85,000. This sample will be equally divided between the 34 sampled communities, that is, 2500 individuals in each community. Considering a potential non-response rate of 30% [34], the baseline recruitment will approach at least 3250 adults in each community. Individuals who are lost to follow-up after enrollment will be replaced annually by other individuals who live in the same community, maintaining the cohort size to a minimum of 2500 individuals at each primary sampling site throughout the 5 years of the project.

### Procedures

Participants will complete online self-report questionnaires about their history and current status and undergo physical and psychological assessments at local community health centers; some of these participants will also receive brain MRI scans at regional general hospitals. Details about the content and method of the evaluations are shown in Table 1.

**Table 1:** Data collected from individual participants

TYPE OF DATA Instrument(s) Collection method	Variables
**DEMOGRAPHIC CHARACTERISTICS** Self-report questionnaire designed by researchers Participants finish the questionnaire online while visiting a local community health clinic	1. **Age:** Age at the day of data collection
	2. **Gender**: Biological sex registered on the ID card (male or female)
	3. **Ethnicity**: Ethnicity registered on the ID card (Han, Miao and so forth)
	4. **Siblings:** Twins, triplets or other siblings with the same parents
	5. **Marital status**: Marriage status at the day of data collection, categorized as single, married, cohabiting with significant other, divorced, widowed, and other
	6. **Education level:** The highest education level, categorized as primary school, middle school, high-school, university and above
	7. **Religion**: Categorized as None, Buddhist, Christian, Islam, Catholicism and other
	8. **Health insurance**: Type of health insurance
	9. **Income**: Family income per year
	10. **Living status:** Living situation such as “where do you live”, “how many roommates” and etc
**PHYSICAL PARAMETERS** Assessed by health workers Participants evaluated at local health clinic	**11. Height**
	**12. Weight**
	**13. Blood pressure**
	**14. Heart rate**
	15. **History of chronic disease**: History of hypertension, diabetes, cardiovascular disease and other chronic disease; specify current treatment (i.e., medications)
	16. **Chronic pain:** Whether suffer from pain in the past or now, location and degree of pain, the influence of pain in daily life and etc
**BIOLOGICAL SAMPLES** Collected by health workers in community All participants provide samples at local health clinic and some participants go to regional hospital to have MRI scan	17. **Blood samples**: 15 ~ 20 ml venous blood obtained through venipuncture by certified nurses
	18. **Urine samples:** 10 ~ 15 ml fresh urine collected by participants under supervision of researchers
	19. **Faeces samples:** 1 g fresh faeces collected by participants under supervision of researchers
	20. **Hair samples:** 20 hairs over 3 cm obtained by researchers
	21. **Brain MRI scan:** MRI scan of whole brain will be performed after evaluation of psychological health. All high-risk participants are referred for a brain MRI scan at a regional hospital, and low-risk and moderate-risk participants are given the option of a referral for a brain MRI scan
**PSYCHOLOGICAL HEALTH** Self-reported questionnaires, and scales completed by local physicians, and diagnostic assessments by participating psychiatrists All participants come to the local clinic to complete the questionnaires online and are assessed by local clinicians and some participants are administered formal diagnostic examination by a participating psychiatrist	22. **Personality traits:** Big Five Inventory scale
	23. **Coping Style**: Simplified Coping Style Scale
	24. **Loneliness:** UCLA Loneliness Scale
	25. **Depression:** PHQ-9 Depression Scale
	26. **Anxiety:** GAD-7 Anxiety Scale
	27. **Social support:** Multidimensional Scale of Perceived Social Support
	28. **Quality of life:** Quality of Life scale
	29. **Sleep quality:** Insomnia Severity Index
	30. **Evaluation performed by community clinicians and psychiatrists**: Community clinicians will assess the psychological health and risks of depression after participants fill out the self-reported questionnaires. Participants classified as high-risk of depression will be assessed by a psychiatrist (using SCID to determine DSM-5 diagnoses) and undergo a brain MRI scan at a regional hospital (see above)
**OTHER MEASURES** Standard questionnaires and self-report questionnaires, some of which are designed by project researchers Participants come to local health clinic and complete the questionnaires online	31. **Occupation:** Occupation type, working intensity, job satisfaction
	32. **Lifestyle:** Smoking, drinking, exercise, habit, pets and etc
	33. **Childhood exposures:** Raised up by who, family background at childhood, abuse or bullying received as a child or adolescent and etc. Additionally, Child Trauma Questionnaire will be used
	34. **Utilization of mental health services:** Knowledge and demand of mental health services, how many times do participants actually use this resource
	35. **Life events:** Major events in life such as divorce, death of close relatives or friends, and etc. Additionally, Life Events Scale will be used
	36. **Childbearing history (for women):** This variable is designed especially for women, including menstruation, pregnancy, miscarriage, and etc

#### Construction of an online project management system

The online management system for the project includes three components, the Manager, Health Worker, and Participant Modules. The *Management Module* is used by national and regional supervisors to assign participants, allocate research tasks, supervise the research process, and conduct quality-control operations. The *Health Worker Module* is used by local clinicians, psychiatrists, lab technicians, MRI technicians, and researchers who obtain community-level parameters to upload the results of clinical, psychiatric and MRI examinations, to report the collection and analytic results of biological samples (blood, urine, faeces, and hair), and to annually upload the community-level parameters for each of the 34 primary sampling sites. The *Participant Module* is used by participants to register for the study to; complete the self-report questionnaires included in the study; get results of clinical, laboratory and MRI assessments; obtain information about depression and other mental disorders; and get suggestions about how to improve their health.

#### Staff training

The study will be cooperatively managed by 17 mental health institutions and universities around the country, each responsible for research work in one urban community and one rural community. To ensure consistency across the 17 sites, standardized national training of participating researchers and psychiatrists was completed prior to data collection. From July 2022 to August 2022, we organized one online and one in-person training session for researcher staff that included introduction to the aims, procedures, quality control processes, and standardized data collection methods using the online management system. From September 2022 to October 2022, all psychiatrists conducting diagnostic examinations for the study were trained to assess DSM-5 diagnoses using the Structured Clinical Interview for Diagnoses using DSM-5 (SCID-5-CV).

#### Enrollment and baseline evaluation

**Fig. 2:**
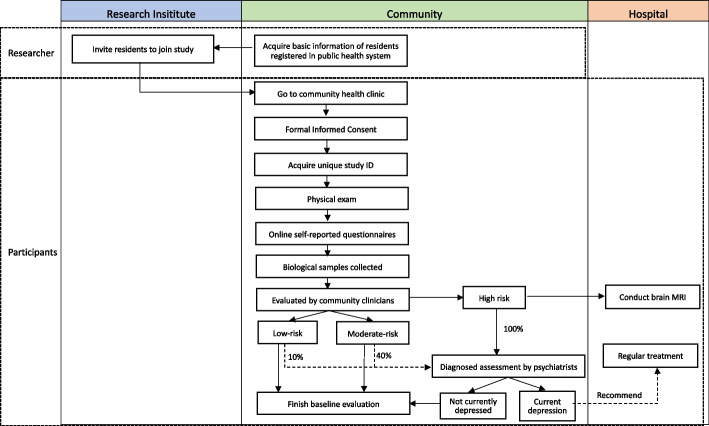
Flowchart of enrollment and baseline assessments

Residents of the 34 primary sampling sites who meet the inclusion and exclusion criteria will be randomly selected from lists of community members registered in the public health system (described above) and invited to participate in the project. To improve the response rate, prior to the enrolment advertising billboards will be set up at the local health service center and messages introducing the study will be sent to all community members via WeChat (social media) site. Enrollment and baseline data collection included the following steps (see Fig. 2):

A) Basic information (e.g., name, age, address, phone number) of residents in the community is extracted from the community health service center (which typically have a registry of virtually all permanent residents in each community).
B) Among the permanent residents 18 years of age or older, 3250 individuals will be randomly selected from the registry of residents maintained by the local health authorities.
C) Researchers make phone calls to selected residents to introduce the China Depression Cohort Study-I and – if the individual expresses interest in participating – to invite them to the local community health service center for an intake interview. Researchers and community health care workers will make home visits to invite individuals who cannot be contacted by phone.
D) When potential participants visit the community clinic they will be provided with details about the project and asked to sign a written informed consent.
E) Baseline assessments of consenting participants are typically conducted at the local health clinic on the same day as enrollment. They include the following:
  - Community clinic physicians conduct a physical exam, measuring weight, height, heart rate and blood pressure.
  - Research nurses collect biological samples of blood, urine and faeces.
  - Trained researchers instruct participants in the use of the online Participant Module and supervise their completion of self-report questionnaires about their demographic characteristics, environmental exposures, and mental health symptoms.
  - Community clinic doctors conduct a psychological evaluation using the expanded version of the 12-item General Health Questionnaire (GHQ-12) [34, 35], which consists of the original Chinese version of the GHQ-12, and 12 additional items to improve its sensitivity. The 12 supplemental items include 6 items described in previous studies [34, 36] (respondents' subjective report of their physical health in the past month; respondents' subjective report of their psychological health in the past month; obsessive thoughts or compulsive behaviors in the last month; restriction of activities because of phobias in the past month; feelings of extreme nervousness or anxiety in the last six months; and social problems due to drinking in the last year), and 6 items developed for the current project based on expert consultation (feeling depressed or down for at least two weeks; seeking help from others due to psychological problems; hospitalization due to psychological problems; suicidal behaviors such as self-poisoning or self-injury; intention of self-harm or suicide; family history of mental disorders such as depression). The 12 GHQ-12 items are each scored as 0 (not present or mild) or 1 (present to a moderate or significant degree); so the total GHQ-12 score ranges from 0 to 12. Each participant will be classified as low-risk of depression (a GHQ-12 score of 0 to 1 and none of the 12 additional items present), moderate-risk of depression (a GHQ-12 score of 2 to 4 and none of the 12 additional items present), or high-risk of depression (a GHQ-12 score of at least 5 or a positive score on any of the 12 additional items) [36].
  - Formal diagnostic examination by a psychiatrist using the SCID-5-CV interview schedule will be conducted in all participants classified as high-risk of depression, in 40% participants classified as moderate-risk of depression and in 10% participants classified as low-risk of depression. Similar to other two-stage epidemiological studies [34, 36], higher proportions of individuals at higher risk of depression are administered the definitive diagnostic interview; this method allows for an efficient use of the limited number of psychiatric personnel available while allowing for accurate prevalence estimates for the total population (by estimating the rates of false positive GHQ-12 rates in the high-risk group and rates of false negative GHQ-12 rates in the moderate- and low-risk groups).
  - All high-risk participants are referred for a brain MRI scan at a regional hospital, and low-risk and moderate-risk participants are given the option of a referral for a brain MRI scan.

#### Follow-up

The initial duration of follow-up is five years. (The study organizers will subsequently apply for additional funding to transform the study into a continuing cohort project.) The follow-up procedures will be as follows:

A) Community-level variables shown in Table 2 (including climatic, environmental and socio-economic characteristics) will be collected annually from national databases for each of the 34 primary sampling sites.
B) Participants who do not have any current or past mental disorder (based on the SCID-5-CV interview) at baseline or at subsequent assessments, the following follow-up assessments will be made *annually*.
  - Information about participants’ diagnoses and treatment of diseases in the prior year will be collected in three ways: review of records of the national medical insurance system (which covers all permanent residents); self-reported information about medical treatment for mental disorders; and results of the annual GHQ-12 screening and SCID-CV interviews.
  - Community clinic physicians conduct a physical exam, measuring weight, heart rate and blood pressure.
  - Research nurses collect biological samples of blood, urine and faeces from a sample of 5% of the participants.
  - Repeat MRI scans we be conducted in each participant who had MRI scan at baseline.
  - Online questionnaires will be sent to participants through e-mail or WeChat. Participants unable to use this online system will be invited to visit the local health clinic where they will be given equipment and any assistance needed to complete the questionnaires.
  - Trained researchers will make phone calls to those who don’t respond to the online questionnaires and to those who self-report as being at high risk of a depressive disorder at any time over the prior year. Participants classified by the researcher as ‘high-risk of depression’ at any time in the prior year will be encouraged to attend an appointment with a psychiatrist at the community clinic or local general hospital.
  - Psychiatrists will perform SCID-5-CV diagnostic assessments of the invited participants who agree to the interview.
C) For participants diagnosed with current or past depressive disorder or other psychiatric disorder based on SCID-5-CV interviews by a psychiatrist at baseline or during follow-up assessments the following follow-up assessments will be conducted.
  - Every 3 months, telephone calls will be performed by trained researchers to check their depressive symptoms, thoughts of suicide or self-harm, suicide attempts or other self-harm behaviors, social functioning, and mental health treatments over the prior 3 months. Individuals classified by the researcher as ‘high-risk of depression’ who have never been diagnosed with a depressive disorder (only had other diagnoses) or have fully recover from a prior episode of depression (i.e., at least 2 months of no clinically significant symptoms) will be encouraged to attend an appointment with a psychiatrist at the community clinic or local general hospital.
  - A psychiatrist will conduct a repeat diagnostic exam (using the SCID-5-CV interview) with invited participants who agree to the interview to determine whether a new-onset major depressive disorder has occurred in the prior 3 months.
  - Each year information about participants’ diagnoses and treatment of diseases (including treatment for mental disorders) from hospital records will be collected from regional hospitals and from the national public health system.
  - Each year community clinic physicians conduct a physical exam, measuring weight, height, heart rate and blood pressure.
  - Each year the standard online questionnaire will be sent to these participants through e-mail or WeChat. Participants unable to use this online system will be invited to visit the local health clinic where they will be given equipment and any assistance needed to complete the questionnaires.
  - Each year research nurses collect biological samples of blood, urine and faeces from participants with a current or lifetime depressive disorder (but NOT from individuals who only have other, non-depressive, mental disorders).
  - Each year, participants with a current or lifetime depressive disorder (but NOT from individuals who only have other, non-depressive, mental disorders), are referred for a brain MRI scan at a regional hospital.

**Table 2:** Environmental level variables at each of the 34 study communities

TYPE OF DATA Source of data Collection method	Variables
**CLIMATE** Official website of China Meteorological Administration Researchers record data for the 34 sites each season each year	1. **Temperature:** Lowest, highest, and average temperature per season
	2. **Precipitation:** Lowest, highest, and average precipitation per season
	3. **Sunshine:** Lowest, highest, and average time of sunshine per season
	4. **Climate alert:** Types and frequencies of climate alerts per season
	5. **Natural disasters**: Intensity and duration of natural disasters, including floods, forest fires, earthquakes, mud-rock flow, and typhoons
**NATURAL ENVIRONMENT** Official website and annual report of Ministry of Ecology and Environment Researchers record data for the 34 sites each season each year	6. **Noise:** Days noise level exceeds standard level, average intensity of noise, type of noise
	7. **Atmospheric environment:** Lowest, highest and average level of air pollution index (e.g., PM 2.5, SO_2_), average days air quality rated as ‘good’ or better
	8. **Water environment:** Water quality and nutrition index of main rivers and lakes
	9. **Soil environment:** Amount and quality of cultivated area
	10. **Radiation:** Intensity of ionizing radiation and electromagnetic radiation
	11. **Pollution:** Amount and type of waste discharged into the environment, including but not limited to solid waste, car exhaust, industrial waste
**SOCIOECONOMIC CHARACTERISTICS** Official website of National Bureau of Statistics and China Statistical Yearbook Researchers calculate socioeconomic indexes based on data provided in the two sources	12. **GDP index:** Total and per capita GDP
	13. **Aging index:** Proportion of population aged over 60 years old
	14. **Poverty gap** Proportion of population living below local poverty level

#### Protection of the rights and health of participants

This is a prospective observational cohort study, so researchers conducting the study will not provide medication or other treatment to participants diagnosed with depression or other mental disorders as part of the study. However, the project will include the following steps to protect the rights and health of participants.

A) Fully informed consent. Prior to providing written informed consent to participate in the study, potential participants and their family members will be fully informed about the expectations and potential risks of the study and about their right to drop out of the study at any time for any reason.
B) At the time of the diagnostic examination, the examining psychiatrist will inform the participant of the diagnostic result of the exam. Basic information about the symptoms, course and appropriate treatment of the condition will be provided to the participant in a non-technical manner that they can easily understand.
C) Participants with current mental disorders who are not receiving treatment will be advised to seek treatment and information about the nearest facilities where they can get mental health treatment will be provided.
D) Self-management manuals about depression: manuals containing knowledge about depressive disorders and self-management of depressive moods will be provided.
E) Participants will be provided with contact information of study researchers that they can use to contact researchers if they feel depressed or need help.
F) For participants considered at high risk of suicide or self-harm, after signing a specific consent form with the participant and his/her family members, the situation will be discussed and the family members will be informed about steps to take to reduce the risk of suicide or self-harm and what they should do and where to seek help if the participant has suicidal or self-harm behavior.
G) Standardized management procedure to deal with suicide and self-harm: Our study has standard regulations (approved by the ethical committee to at the Second Xiangya Hospital of Central South University) to deal with suicide and self-harm behaviors of participants.

#### Outcome measurements

The primary outcome of this study is depression. At recruitment, local community doctors will evaluate the mental health of participants, and refer those considered at high-risk for depression (and some of those considered at moderate- or low-risk for depression) to a psychiatrist who will administer the Structured Clinical Interview for DSM-5-CV to determine whether the individual meets the diagnostic criteria of a depressive disorder. At each annual assessment, trained researchers will screen participants through phone calls and self-reported questionnaires, and refer those considered at high-risk of depression to a psychiatrist for a formal diagnostic assessment.

Secondary outcomes include fatal and non-fatal suicidal behavior, other self-harm behaviors, relapses of depression after recovery from a previous episode, and the prevalence and incidence of other types of mental disorders assessed when administering the SCID-5-CV.

### Data storage and analyses

#### Data storage

The specific individual-level and community-level data collected in the study are shown in Tables 1 and 2, respectively.

Participants will be assigned a unique study identification number that will used when recording all individual-level data about the participant. To protect the identify of participants, all identifying information about participants (name, national ID number, telephone number, date of birth, address, etc.) will not be included in electronic databases for the project but, rather, kept in paper documents (with the unique study identification number) held in a secure location at each of the 17 research centers.

All paper-based forms and all data directly entered on computers or tablets at the local clinics or the regional hospitals will be safely transferred to secure locations or password-protected computers at the coordinating research centers as soon as is practical. All data recorded on paper forms will be checked by two researchers prior to entry on a database to minimize missing data. At each of the 17 research locations a standalone password protected computer will be used to enter and store hardcopy coded data and to store all softcopy data. All data will be stored and preserved in a designated secured and locked space, available only to the assigned data manager and his/her assistants at each research site.

#### Storage and primary analyses of biological samples and MRI image

A total of 15 ~ 20 ml venous blood, 10 ~ 15 ml fresh urine, 1 g fresh faeces and 20 hairs over 3 cm will be obtained from each participant. These biological samples will be centrifuged at local laboratory and aliquoted for long-term storage in ultra-cryogenic refrigerator of -80 °C. Part of the blood, hair, urine and stool samples will be used for biochemistry, genomics, metabolomics and gut microbiome analysis. The residual sample materials will be carefully stored and analyzed when needed.

All raw images from MRI scans will be transferred to the Second Xiangya Hospital, where analyses of images, data processing and storage will be performed. High resolution structural 3D-T1 images will be collected and brain morphology including whole brain volume, gray matter volume, white matter volume, thickness, and surface area will be assessed. Resting-state fMRI images will be used to describe the spontaneous functional activity and connectivity of neurons. Diffusion tensor imaging (DTI) data will be used to assess the microstructure of white matter. Data from these primary analyses will be uploaded into the management system and stored at the local password protected computer. In the future, these raw data will be available for additional analyses.

#### Data analyses

Three main analyses will be conducted. P values of < 0.05 will be considered statistically significant.

The first set of analyses will describe the characteristics of participants and the detailed epidemiology of depression, including the prevalence, incidence, regional characteristics, and trends in the demographic characteristics of depression over time. Means and standard deviations will be used to describe continuous variables, and numbers and percentages will be used to describe categorical variables. Poststratification weights will be used to estimate the national prevalence and incidence of depression. Latent variable analyses and disease trajectory analyses will be used to describe the changes of depressive disorders over time.

The second set of analyses will 1) identify risk factors that influence the onset, severity and course of depressive disorders; 2) identify characteristics that can help distinguish distinct subtypes of depression; and 3) assess potential pathophysiological mechanisms that link the different types of factors associated with depression – external environmental factors, social factors, psychological factors, and biological factors. T-tests, analyses of variance, Kruskal–Wallis tests and Chi-square tests will be used to analyze differences between groups. Correlation analyses will be used to check correlations between variables. To identify potential risk factors for depression, Cox regression analyses will be used to estimate the hazard ratios (HRs) of the association of specific bio-psycho-social risk factors with the diagnosis of depression. Logistic and liner regression will be used to identify factors associated with the severity or depressive episodes. Genomic analysis (including GWAS analysis, Mendelian Randomization), metabolomic analysis and gut microbiome analysis may also be used to explore potential biological mechanisms of depression.

The third set of analyses will use the findings about risk factors to generate and test different prediction tools of depression. Different predictive models will be generated using machine learning algorithms, including Support Vector Machine, Decision Tree and Random Forest algorithms [37, 38]. Performance of the different models will be compared using the area under the receiver operating curve, prospective prediction results (sensitivity, specificity, accuracy, positive predictive value, negative predictive value) and decision curve analysis (DCA) [38].

## Quality control

The members of the quality control committee include the lead collaborators and study managers at the 17 institutes, senior researchers on the project’s consultative committee, and staff members of the ethics committee at the Second Xiangya Hospital, Central South University. The Second Xiangya Hospital, Central South University is the lead institute of the China Depression Cohort Study-I, responsible for study design, national training, quality control, database management and primary data analyses.

The study procedures comply with national policies regarding the requirements of human studies. To ensure the consistency of the study in different settings, local pilot studies will be conducted to assess the feasibility of the proposed study procedures at all 34 primary sampling sites, and the procedures will be revised as needed based on the findings of the pilot studies. The specific goals of the pilot studies are to 1) identify methods for increasing enrollment and retention of participants, 2) develop methods for helping elderly participants complete online assessments, 3) test the effectiveness of the quality control and data entry procedures, 4) ensuring that sufficient numbers of rural participants (who may live far from hospitals that conduct MRI assessments) complete the brain MRI scans, and 5) develop uniform parameters for conducting the brain MRI scans at all participating general hospitals.

Once the formal study starts, local compliance with study procedures will be assessed during quarterly online meetings of the quality control committee to discuss and resolve problems that arise. Data collected at the 34 primary sampling sites will be checked in real time using the online project management system. Irregular, semiannual on-site inspections by project consultants and technical staff from the quality control committee will assess local study progress, observe the sample recruitment and data collection procedures, and check the methods of data entry, storage, and analyses.

## Ethics and dissemination

The China Depression Cohort Study-I will be carried out in accordance with the principles and specifications published by Chinese government in collecting, storing, managing and using data from human subjects [39]. Ethics approval (NO.2022S010) for the overall project has been obtained from the Ethics Committee of National Clinical Medical Research Center, Second Xiangya Hospital, Central South University. Separate ethics approval will also be obtained from the 17 research centers (responsible for one rural site and one urban site) participating in the project.

Each participant will be informed about the study objectives and procedures and told that they can withdraw from the study at any time for any reason. They will also be given the option to 1) permit the use of their coded (de-identified) human biological samples in future studies, 2) consent to being contacted in the future for follow-up assessments, intervention studies, and other research projects. They will be asked to sign a written informed consent after they understand and agree to participate.

Results from this study will be disseminated by publication of peer-reviewed manuscripts and by presentations at scientific meetings and conferences. The researchers may also communicate with other researchers worldwide through meetings and other events and promote the results to the public using media releases.

## Discussion

The onset and course of depression are determined by complex interactions between an individual’s genes and the environment in which the individual exists. Many studies support this hypothesis. 1) Gut microbiota – a crucial part of the body’s microenvironment – is associated with depression and anxiety via microbiome-gut-brain pathways [40], and may also be related to the communication about peripheral inflammation to the brain [41]. 2) An inappropriate rearing environment, particularly a setting that includes childhood maltreatment, increases the risk of childhood depression [42, 43]. 3) Housing instability can result in higher rates of depression in mothers experiencing a non-marital birth [44]. And 4) Employment and work environments [45], early life stress [46], and other social environmental factors influence the development of major depression.

Despite the extensive research in this space, systematic reviews and meta-analyses about the cause and course of depression report heterogeneous results and often failed to adjust the findings for important confounding factors [7, 47]. The specific biomarkers and environmental markers of depression remain unknown. Large, longitudinal studies with community-based samples that simultaneously consider the multiple interacting factors which influence the onset and course of the disorder are needed. The China Depression Cohort Study-I will attempt to achieve this objective. Collecting a comprehensive set of both individual-level and community-level variables over time, we plan to reframe the understanding of depression from a ‘biology-psychology-society’ perspective. We believe that this perspective will help psychiatrists better understand depression and, thus, develop more effective depression subgroup-specific antidepressant drugs and other interventions based on the new biomarkers and relationships identified in the study. The study will establish a comprehensive dataset containing clinical and biological samples for studies of depression by collaborators around the world.

The study also has some limitations. First, this study hasn’t covered every province, autonomous region and municipality in China. Second, the practicalities of identifying such a large cohort, conducting extensive evaluations of participants, and following participants for a minimum of 5 years required some trade-offs between the feasibility of conducting the project and the random selection of study sites. The selection of DSP within provinces and the selection of urban and rural communities within DSP was not strictly random, DSP and communities with very limited health care resources (that would be unable to manage the project) and communities with very small populations (that would not be able to recruit the minimum sample of 2500 individuals) were not included in the sampled DSP and communities. Third, the China Depression Cohort Study-I only considers depression among adults. The parallel China Depression Cohort Study-II will include middle school students 12 ~ 18 years of age, and we hope to subsequently expand the cohort to include children under 12 years of age and to continuously follow cohort participants of all ages.

## Current status of the study

This study has developed the instruments to be employed in the project, identified the collaborating sites and personnel, provided national staff training, and constructed and tested the online project management and data recording systems. Preliminary pilot studies in several communities showed that it takes 1.5 ~ 2 h to complete the baseline procedures; 100% of participants reported being satisfied with the procedures and 94% reported being willing to accept follow-up assessments. The local pilot studies at all 34 primary sampling sites began in March 2023.

## Data Availability

Not Applicable.

## Abbreviations

DALYs: Disability-adjusted life-years
CRP: C-reaction Protein
PRS: Polygenic Risk Score
NESDO: The Netherlands Study of Depression in Older Persons
NEMESIS: The Netherlands Mental Health Survey and Incidence Study
CHCCS: The China Hainan Centenarian Cohort Study
CKB: The China Kadoorie Biobank
DSM-5: Diagnostic and statistical manual of mental disorders
SCID-5-CV: The Structured Clinical Interview for Diagnoses using DSM-5
MRI: Magnetic Resonance Imaging
GHQ-12: The General Health Questionnaire 12
DTI: Diffusion tensor imaging
fMRI: Functional magnetic resonance imaging
DCA: Decision curve analysis
